# Role of Zinc and Clinicopathological Factors for COVID-19-Associated Mucormycosis (CAM) in a Rural Hospital of Central India: A Case-Control Study

**DOI:** 10.7759/cureus.22528

**Published:** 2022-02-23

**Authors:** Sunil Kumar, Sourya Acharya, Shraddha Jain, Samarth Shukla, Dhruv Talwar, Divit Shah, Vidyashree Hulkoti, Sana Parveen, Mansi Patel, Sujal Patel

**Affiliations:** 1 Department of Medicine, Jawaharlal Nehru Medical College, Datta Meghe Institute of Medical Sciences (Deemed to be University), Wardha, IND; 2 Department of Otorhinolaryngology, Jawaharlal Nehru Medical College, Datta Meghe Institute of Medical Sciences (Deemed to be University), Wardha, IND; 3 Department of Pathology, Jawaharlal Nehru Medical College, Datta Meghe Institute of Medical Sciences (Deemed to be University), Wardha, IND; 4 Department of Otolaryngology, Jawaharlal Nehru Medical College, Datta Meghe Institute of Medical Sciences (Deemed to be University), Wardha, IND

**Keywords:** central india, rural hospital, zinc, mucormycosis, covid-19

## Abstract

Introduction

Coronavirus disease 2019 (COVID-19)has been a difficult enemy to beat for healthcare professionals around the world. However, even before the end of the COVID-19 pandemic, there has been an emergence of a new combatant in the form of opportunistic fungal infections with a high rate of morbidity and mortality, creating havoc throughout the globe.

Methods

A case-control single-center study was conducted in Datta Meghe Institute of Medical Sciences, Wardha, Maharashtra. All the subjects who were included in the study were tested positive for COVID-19 through the reverse transcriptase-polymerase chain reaction (RT-PCR) method and the cases were defined as patients with biopsy-proven mucormycosis, whereas control were subjects who did not develop mucormycosis. The duration of the study was three months, from June 2021 to August 2021.

Result

A total of 55 cases and 50 controls were enrolled in the study. The use of zinc was found to be significantly associated with COVID-19-associated mucormycosis, with 89.1% of the cases having a history of zinc intake and only 52% of controls having a history of zinc intake( p-value <0.001). Diabetes mellitus was found to be significantly associated with COVID-19-associated mucormycosis with 83.6% of the cases and 16% of the controls having diabetes mellitus (p-value <0.001). Although the use of steroids in cases was more with 98.2% of the cases and 54% of the control receiving steroids; this difference was not significant statistically (p-value of 1.00).

Conclusion

We conclude that apart from diabetes mellitus and other immunosuppressive states, zinc might be the hidden culprit behind the sudden surge of COVID-19-associated mucormycosis worldwide owing to the self-administration of zinc by the patients to acquire innate immunity and over-prescription of multivitamins by the treating clinicians. However, this association required further studies in order to be proved.

## Introduction

Coronavirus disease 2019 (COVID-19) has swept the entire world and created a worldwide alarm for healthcare professionals. Even though most of the patients with COVID-19 experience mild to moderate form of illness primarily affecting the respiratory system, individuals with co-morbidities and the elderly are at greater risk of contracting a severe form of the disease. Infection in these cases shows a rapid course, which may lead to clinical deterioration of the patient causing acute respiratory distress syndrome [1]. Co-infection from bacterial as well as fungal pathogens is well-reported previously in patients who suffered from influenza, Middle East Respiratory Syndrome (MERS), and severe acute respiratory syndrome (SARS). However, knowledge of co-infections, especially fungal infections in patients suffering from COVID-19 remains limited owing to the short course of time since the emergence of this infection [2].

Hence, the frontline clinicians battling the ongoing pandemic of COVID-19 need to be aware of the predisposing factors that lead to the contraction of fungal infections in COVID-19 patients. Patients who develop acute respiratory distress syndrome, receive a high dose of steroids, broad-spectrum antibiotics, and immunomodulators, and need mechanical ventilation are at greater risk of contracting aspergillosis, candidiasis, mucormycosis, and Pneumocystis jiroveci pneumonia [3].

There is a sparsity of reported data on COVID-19-associated mucormycosis. Hence, we conducted a study to determine the clinicopathological factors and possible role of oral zinc administration in the development of COVID-19-associated mucormycosis.

## Materials and methods

**Figure 1:**
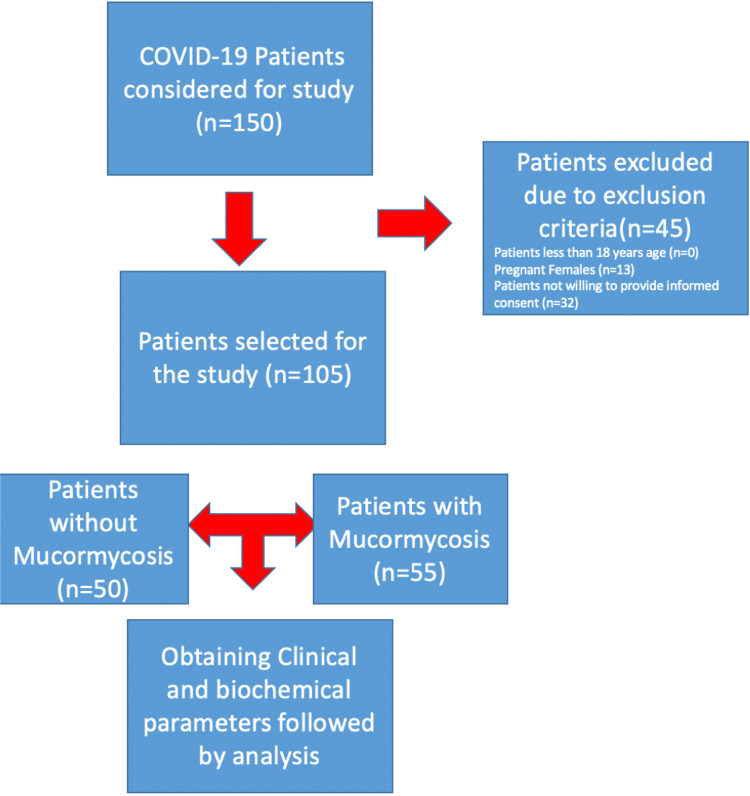
Flowchart depicting the methodology of the study

This case-control study was conducted in the Department of Medicine, Datta Meghe Institute of Medical Sciences, Wardha, Maharashtra, India, from June 2021 to August 2021 after obtaining ethical clearance from the institutional ethics committee with ethical clearance number DMIMS(DU)/IEC/2021/338. Inclusion criteria were COVID-19 patients who tested positive by RT-PCR) aged 18 years or above. Patients with mucormycosis and COVID-19 positive were included in the case group and those without mucormycosis infection but with COVID-19-positive status were included in the control group. Exclusion criteria were patients less than 18 years, pregnant females, and patients not willing to give informed consent. A total of 150 patients admitted for COVID-19 infection were considered for this study. After applying exclusion criteria, a total of 105 patients meeting the inclusion criteria were included in the study after obtaining proper informed consent. All inflammatory markers were sent during the first 24 hours of admission and close follow-up, and signs and symptoms for mucormycosis were noted and tested for this fungal infection. Analysis of complete blood count was done through the DxH 800 hematology analyzer (Beckman Coulter, South Drive, IN) while all inflammatory markers, renal function test, and liver function test were done through Vitros 5600 (Ortho Clinical Diagnostics, Raritan, NJ). High-resolution computed tomography (HRCT) was conducted for all the patients within 48 hours of admission. All patients were asked about the consumption of zinc tablets prior to admission and the dosage and duration for the same. All enrolled patients were screened for co-morbidities on the basis of detailed history-taking about any known previous illness and screening for various diseases, including chronic obstructive pulmonary disease on the basis of a chest X-ray, ischemic heart disease based on electrocardiography, diabetes mellitus based on glycated hemoglobin (HbA1c), obesity on the basis of body mass index, and hypertension on the basis of blood pressure, all recorded within 24 hours of admission. All the patients were observed until cure or death and the length of hospital stay was also noted. Figure 1 shows the flowchart of methodology for this study. The primary endpoint of the study was to assess the association of zinc with COVID-19-associated mucormycosis and the secondary endpoint was to analyze the association of diabetes mellitus with COVID-19-associated mucormycosis.

Study definition

Only patients having COVID were enrolled in the study. Patients with mucormycosis were taken in the case group and patients without mucormycosis in the control groups. Both groups were asked in detail about different variables and about zinc medications if they were on any. The diagnosis of mucormycosis was established by tissue biopsy and histopathological examination using hematoxylin and eosin (H&E) staining.

Statistical analysis

Descriptive statistics were applied to summarize the demographic data. Results are reported as medians and interquartile ranges or means with standard deviations or counts and frequency. Fisher's exact test and the chi-square test were applied to detect significant differences among stratifications. The software program SPSS v23 (IBM Corp., Armonk, NY) was used to analyze the statistical data.

## Results

A total of 105 patients were included in the analysis. Fifty-five subjects were cases who had COVID-19-associated mucormycosis, whereas 50 subjects were controls who did not have COVID-19-associated mucormycosis. All the 105 subjects were positive for COVID-19. Eighty-one point eight percent (81.8%) of the cases and 60% of controls were male, whereas 18.2% percent of the cases and 40% of controls were females. The mean age of cases was 53.82 ± 10.23 years, whereas the mean age of controls was 55.58 ± 14.53 years. The association of various parameters with cases and controls is shown in Table 1. The following variables were significantly associated (p<0.05) with the variables Group, Gender, DM, HTN, Hemoglobin, Platelet Count, Urea, Sodium, Potassium, ALT (U/L), AST (U/L), ALP (U/L), Uric Acid (mg/dL), D-Dimer, Ferritin, LDH, CRP, Zinc, and Outcome.

**Table 1:** Association between groups and parameters ***Significant at p<0.05, 1: t-test, 2: chi-square test, 3: Wilcoxon-Mann-Whitney U test, 4: Fisher's exact test HRCT-High Resolution Computed Tomography; DM-Diabetes Mellitus; HTN-Hypertension; TLC-Total Leukocyte Count; ALT-Alanine Transaminase; AST-Aspartate Aminotransferase; ALP-Alkaline Phosphatase; LDH-Lactic Acid Dehydrogenase; CRP-C-Reactive Protein; Bipap-Bi-level Positive Airway Pressure

Parameters	Group		p value
	Case (n = 55)	Control (n = 50)	
Age (Years)	53.82 ± 10.23	55.58 ± 14.53	0.478^1^
Gender***			0.013^2^
Male	45 (81.8%)	30 (60.0%)	
Female	10 (18.2%)	20 (40.0%)	
HRCT Score	12.73 ± 3.11	13.80 ± 5.87	0.253^1^
Il-6	79.76 ± 61.76	741.23 ± 1091.80	0.075^3^
DM (Yes)***	46 (83.6%)	8 (16.0%)	<0.001^2^
HTN (Yes)***	31 (56.4%)	12 (24.0%)	<0.001^2^
Hemoglobin ***	9.98 ± 1.35	11.82 ± 2.24	<0.001^3^
TLC	9903.45 ± 5233.19	8528.00 ± 4724.45	0.094^3^
Platelet Count***	2.69 ± 1.00	2.18 ± 0.96	0.002^3^
Urea***	33.53 ± 33.75	43.64 ± 38.05	0.009^3^
Creatinine	1.28 ± 1.14	1.34 ± 1.42	0.348^3^
Sodium***	136.29 ± 5.53	139.66 ± 6.02	0.002^3^
Potassium***	3.96 ± 0.86	4.56 ± 0.74	<0.001^1^
ALT (U/L)***	46.09 ± 158.90	45.37 ± 30.70	0.002^3^
AST (U/L)***	184.04 ± 1094.36	59.59 ± 39.28	<0.001^3^
ALP (U/L)***	163.60 ± 118.67	85.10 ± 50.20	<0.001^3^
Uric Acid (mg/dL)***	4.41 ± 2.01	5.26 ± 2.30	0.035^3^
D-Dimer***	11.44 ± 71.36	1.92 ± 2.22	0.003^3^
Ferritin ***	669.97 ± 342.48	396.41 ± 371.82	<0.001^3^
LDH***	348.11 ± 279.98	616.26 ± 482.96	<0.001^3^
CRP***	105.78 ± 287.11	8.30 ± 7.51	<0.001^3^
Steriod Use (Yes)	54 (98.2%)	27 (54%)	1.000^4^
Duration Of Covid Infection (Days)	16.82 ± 5.55	17.00 ± 11.75	0.636^3^
Ventilatory Support (Yes)	49 (89.1%)	45 (90.0%)	0.879^2^
Ventilatory Support Given			0.124^4^
None	6 (10.9%)	5 (10.0%)	
Oxygen	43 (78.2%)	35 (70.0%)	
BIPAP	6 (10.9%)	5 (10.0%)	
Mechanical Ventilation	0 (0.0%)	5 (10.0%)	
Zinc (Yes)***	49 (89.1%)	26 (52.0%)	<0.001^2^
Outcome***			<0.001^4^
Discharged	46 (83.6%)	47 (94.0%)	
Death	9 (16.0%)	3 (6.0%)	

Table 2 depicts the association of zinc with cases and controls. The chi-square test was used to explore the association between 'Group' and 'Zinc'. There was a significant difference between the various groups in terms of the distribution of zinc (χ2 = 17.655, p = <0.001). The strength of association between the two variables (Cramer's V) was 0.41, showing moderate association, whereas the strength of association between the two variables (Bias Corrected Cramer's V) was 0.4, showing moderate association. Eighty-nine point one percent (89.1%) of the participants in the case group had zinc consumption. Ten point nine percent (10.9%) of the participants in the case group did not receive zinc. Fifty-two percent (52.0%) of the participants in the control group had received zinc. Forty-eight percent (48.0%) of the participants in the control group did not receive zinc as shown in Table 2. Therefore, participants in the case group Group had the larger proportion of subjects with zinc intake, whereas participants in the group control Group had the larger proportion of subjects who did not receive zinc.

**Table 2:** Association between group and zinc (n = 105) Case - Patients with COVID-19-associated mucormycosis Control - Patients with COVID-19 without COVID-19-associated mucormycosis

Zinc	Group			Chi-Squared Test	
	Case	Control	Total	χ2	P-Value
Yes	49 (89.1%)	26 (52.0%)	75 (71.4%)	17.655	<0.001
No	6 (10.9%)	24 (48.0%)	30 (28.6%)		
Total	55 (100.0%)	50 (100.0%)	105 (100.0%)		

Fisher's exact test was used to explore the association between outcome and subjects as well as controls. There was a significant difference between the various groups in terms of distribution of outcome (χ2 = 12.158, p = <0.001). The strength of association between the two variables (Cramer's V) was 0.34 showing moderate association. The strength of association between the two variables (Bias Corrected Cramer's V) was 0.33 showing moderate association. Eighty-three point six percent (83.6%) of the participants in the case group had the outcome as discharged or treated, whereas 16.0% of the participants in the case group Group had the outcome as death. Ninety-four percent (94.0%) of the participants in the control group had the outcome as discharged, whereas 6.0% of the participants in the control group had the outcome as death. Participants in the case group had a larger proportion of expired patients, whereas participants in the control group had the larger proportion of discharged patients as shown in Table 3.

**Table 3:** Association between group and outcome (n = 105) Case-Patients with COVID-19 associated mucormycosis; Control-Patients with COVID-19 without COVID-19-associated mucormycosis

Outcome	Group			Fisher's Exact Test	
	Case	Control	Total	χ2	P Value
Discharged	46 (83.6%)	47 (94.0%)	93 (88.5%)	12.158	<0.001
Death	9 (16.0%)	3 (6.0%)	12 (11.4%)		
Total	55 (100.0%)	50 (100.0%)	105 (100.0%)		

Fisher's exact test was used to explore the association between outcome and zinc intake, as more than 20% of the total number of cells had an expected count of less than 5. There was no significant difference between the various groups in terms of the distribution of zinc (χ2 = 0.066, p = 0.771) as shown in Table 4. The strength of association between the two variables (Cramer's V) = 0.03 showing little or no association. The strength of association between the two variables (Bias Corrected Cramer's V) = 0 showed little or no association. Seventy-one point nine percent (71.9%) of the participants in the group of discharged subjects had zinc intake, whereas 28.1% of the participants in the group of discharged subjects had no history of zinc intake. Sixty-eight point eight percent (68.8%) of the participants in the group of expired subjects had zinc intake, whereas 31.2% of the participants in the group of expired patients had no history of zinc intake.

**Table 4:** Association between outcome and zinc (n = 105)

Zinc	Outcome			Fisher's Exact Test	
	Discharged	Death	Total	χ2	P Value
Yes	69 (71.9%)	6 (68.8%)	75 (71.4%)	0.066	0.771
No	27 (28.1%)	3 (31.2%)	30 (28.6%)		
Total	96 (100.0%)	9 (100.0%)	105 (100.0%)		

## Discussion

There has been an increase in the reporting of mucormycosis in COVID-19 patients, raising concern for healthcare professionals in identifying and eliminating the probable factors responsible.

The prevalence of mucormycosis is still under study; however, it is reported as 1.8% and 3.36% from two studies [4-5]. However, in a multicenter study conducted in India, COVID-19-associated mucormycosis had a prevalence of 65.2% among mucormycosis patients.

Corticosteroid and diabetes mellitus have been established as risk factors for fungal infections such as mucormycosis. Corticosteroids lead to immunosuppression, which, in turn, leads to opportunistic infections. Diabetes mellitus, on the other hand, leads to hyperglycemia causing glycation of iron-sequestering protein and release of free iron. There is upregulation of glucose-regulated protein 78 (GRP-78) receptor and increased expression of spore coat protein homologs (CotH). This, in turn, leads to increased fungal binding as well as invasion.

In a study conducted by Gupta et al. in multiple centers of India, it was observed that a total of 115 patients with COVID-19-associated mucormycosis were identified and all of them had received corticosteroids, and 85.2% of the patients were diabetic [6]. These findings were similar to our study where 54 patients (98.2%) of the control group had received steroids and 46 patients (83.6%) were diabetic. However, mortality was reported in 25 patients (21.7%), which was more than the finding of our study where morality in the case group was 16.0%.

It is interesting to note that most of the cases of mucormycosis associated with COVID-19 have been reported from India. A systematic review conducted by Singh et al. had reported the prevalence of steroid use in COVID-19-associated mucormycosis to be 76% and diabetes mellitus in 80% of the cases [7]. A retrospective study carried out in India by Sen et al. reported that steroid use was present in 87% of patients, whereas diabetes was present in 79% of the patients with COVID-19-associated mucormycosis [8].

In a study conducted by Mishra et al., it was found that average glycosylated hemoglobin levels in patients with COVID-19-associated mucormycosis were 9.06% [4].

Hence, diabetes mellitus, which was found to be significantly associated with COVID-19-associated mucormycosis in our study, has been previously studied and found to be an important predisposing factor for COVID-19-associated mucormycosis by other studies as well, especially in India. However, even though a large number of cases (98.2%) received corticosteroids when compared to control (54%) in our study, it was statistically not significant.

Another aspect of our study is the role of zinc intake in developing COVID-19-associated mucormycosis. In a review conducted by Kehl et al., it was reported that fungal cells must acquire zinc in order to develop properly and complete their life cycle even during their existence as saprophytes or during the infection phase. Therefore, in order to prevent fungal invasion, mammals reduce levels of free zinc and other metals in the body [9].

In a study conducted by Muthu et al. in Chandigarh (India), it was found that zinc promoted the in vitro growth of some of the isolates of Rhizopus arrhizus. It was postulated that an increase seen in the biomass of fungus along with zinc levels might promote the development of COVID-19-associated mucormycosis in immunocompetent individuals [10]. Along with the experimental aspect, an interesting inclusion in this study was the clinical aspect, with cases and control enrolled to look for features of COVID-19-associated mucormycosis. It was found that 26 of the 35 participants had not received supplementation with zinc. Only six of the cases and three of the controls had received zinc supplementation, and this difference was not significant when tested statistically.

In our study, 49 of the cases and 26 of the controls had a history of zinc supplementation intake. This difference was found to be significant statistically with a p-value of less than 0.001.

The outcome was also significantly associated with the case group having a higher proportion of expired patients showing that mucormycosis is further increasing the burden of the pandemic by increasing the mortality. Hence, COVID-19-associated mucormycosis required immediate attention from clinicians in order to reduce COVID-19-related deaths worldwide.

There are currently a number of trials going on, which are studying the antiviral effects of zinc and its benefits against COVID-19. Pal et al., in their review of zinc and its action on viral infection, stated that the anti-inflammatory and antioxidant properties of zinc may be beneficial while managing COVID-19 [11]. In a review conducted by Alexander et al., it was reported that assessing nutritional deficiencies, including zinc levels in COVID-19 patients, is essential as the early implementation of zinc in COVID-19 might alter the severity of the disease [12]. In an observational study conducted by Jothimani et al., it was concluded that COVID-19 patients with a deficiency of zinc had poor outcomes [13]. However, in the COVID A to Z trial, it was found that a high dose of zinc gluconate, ascorbic acid, or a combination of the two supplements did not significantly alter the course of COVID-19 infection [14].

Zinc chelators include clioquinol, phenanthroline,ethylenediaminetetraacetic acid (EDTA), ethylene glycol-bis(2-aminoethylether)-N,N,N′,N′-tetraacetic acid (EGTA), and 1,2-bis(o-aminophenoxy)ethane-N,N,N′,N′-tetraacetic acid (BAPTA). In a study conducted by Leonardelli et al., the in vitro activity of combinations of zinc chelators with amphotericin B and posaconazole was tested against six Mucorales species [15]. It was reported that posaconazole with clioquinol showed promising results, especially against Rhizopus microsporus, however, the results of amphotericin B with zinc chelators were discouraging.

Zinc can, therefore, be a dual-edged sword in the management of COVID-19, which should be used judiciously. However, further studies are indeed required to establish this association of zinc along with mucormycosis, which may help drive the management of COVID-19-associated mucormycosis with the use of zinc chelators.

Presently, many studies have been conducted to detect potent markers of severity for COVID-19 [16-18] and the effect of diabetes mellitus on COVID-19, leading to opportunistic infections [19]. However, there is a sparsity of data to determine important factors like zinc for COVID-19-associated opportunistic infection, such as mucormycosis, making the topic of this study important for public health.

Limitation

Our study was carried out in a rural hospital in central India, therefore due to monetary issues and non-availability of equipment, serum zinc levels could not be elicited in our subjects, which would have been ideal to establish an association of zinc with mucormycosis. Also, another limitation of this study is that this was a single-center study with no representation from other regions of India.

## Conclusions

We conclude that diabetes mellitus is a significant risk factor for developing COVID-19-associated mucormycosis, and hence the treating clinicians should be vigilant while prescribing corticosteroids or immunomodulators to such patients and should monitor such patients regularly for the symptoms and signs of mucormycosis. Intake of zinc was also found to be associated significantly with COVID-19-associated mucormycosis, therefore treating clinicians should restrict the use of multivitamins containing zinc and other metals, which might promote fungal growth. However, further studies in order to establish an association of zinc and COVID-19-associated mucormycosis and to explore the use of zinc chelators in mucormycosis are required.
